# Substrate roughening improves swimming performance in two small-bodied riverine fishes: implications for culvert remediation and design

**DOI:** 10.1093/conphys/cox034

**Published:** 2017-05-26

**Authors:** Essie M. Rodgers, Breeana M. Heaslip, Rebecca L. Cramp, Marcus Riches, Matthew A. Gordos, Craig E. Franklin

**Affiliations:** 1 School of Biological Sciences, The University of Queensland, Saint Lucia, Brisbane, Queensland 4072, Australia; 2 Department of Primary Industries, 1243 Bruxner Highway, Wollongbar, New South Wales 2477, Australia

**Keywords:** culvert design, passage, turbulence, velocity barrier

## Abstract

Worldwide declines in riverine fish abundance and diversity have been linked to the fragmentation of aquatic habitats through the installation of instream structures (e.g. culverts, dams, weirs and barrages). Restoring riverine connectivity can be achieved by remediating structures impeding fish movements by, for example, replacing smooth substrates of pipe culverts with naturalistic substrates (i.e. river stones; culvert roughening). However, empirical evaluations of the efficacy of such remediation efforts are often lacking despite the high economic cost. We assessed the effectiveness of substrate roughening in improving fish swimming performance and linked this to estimates of upstream passage success. Critical swimming speeds (*U*_crit_) of two small-bodied fish, purple-spotted gudgeon (*Mogurnda adspersa*; 7.7–11.6 cm total length, BL) and crimson-spotted rainbowfish (*Melanotaenia duboulayi*; 4.2–8.7 cm BL) were examined. Swimming trials were conducted in a hydraulic flume fitted with either a smooth acrylic substrate (control) or a rough substrate with fixed river stones. Swimming performance was improved on the rough compared to the smooth substrate, with *Mo. adspersa* (*U*_crit-smooth_ = 0.28 ± 0.0 m s^−1^, 2.89 ± 0.1 BL s^−1^, *U*_crit-rough_ = 0.36 ± 0.02 m s^−1^, 3.66 ± 0.22 BL s^−1^, mean ± s.e) and *Me. duboulayi* (*U*_crit-smooth_ = 0.46 ± 0.01 m s^−1^, 7.79 ± 0.33 BL s^−1^; *U*_crit-rough_ = = 0.55 ± 0.03 m s^−1^, 9.83 ± 0.67 BL s^−1^, mean ± s.e.) both experiencing a 26% increase in relative *U*_crit_. Traversable water velocity models predicted maximum water speeds allowing successful upstream passage of both species to substantially increase following roughening remediation. Together these findings suggest culvert roughening may be a solution which allows hydraulic efficiency goals to be met, without compromising fish passage.

## Teaser

Disruption of riverine connectivity by artificial structures, such as culverts, can obstruct critical fish movements. We investigated the effectiveness of replacing smooth substrates with rough, naturalistic substrates (i.e. river stones) in improving fish swimming capacity and modelled fish passage through culverts.

## Introduction

Disruption of riverine connectivity is one of the leading threats to the persistence of riverine fishes (Paul and Meyer, 2001; Nilsson *et al*., 2005; Liermann *et al*., 2012). Instream structures (e.g. dams, weirs, barrages and culverts) can impede up- and down-stream fish movements by creating physical (e.g. dam walls), hydraulic (e.g. excessive water velocities), physiochemical (e.g. low dissolved oxygen) and behavioural (e.g. low light-levels) barriers. Free and efficient movement throughout waterways is essential to the survival and reproductive success of many fishes (Fausch *et al*., 2002). Small-scale, intra-biome movements can be necessary for defending territory, avoiding predators and accessing food (Clapp *et al*., 1990; Harvey, 1991); whereas large-scale, inter-biome movements are often necessary for reaching spawning grounds, habitat selection and maintenance of genetic diversity (Gowan and Fausch, 2002; Yamamoto *et al*., 2004). Artificial structures can disrupt these processes and have been linked to local extinction events globally (Gehrke *et al*., 2002; Quinn and Kwak, 2003; Lundqvist *et al*., 2008).

Fish passes have been developed to facilitate fish movements around instream barriers, but a comprehensive set of conditions conducive to optimizing passage (e.g. water velocity, turbulence and temperature, and structure slope, height and length) is unavailable for many species. Research in this area has predominantly focused on enabling fish to bypass large obstructions such as dams and weirs (Starrs *et al*., 2011; Bunt *et al*., 2012; Li *et al*., 2015). Designing passes at small but numerous barriers, such as culverts, is however receiving increasing attention (Mueller *et al*. 2008; Feurich *et al*. 2012; Rodgers *et al*., 2014).

The combined effect of culverts and other small barriers (e.g. low head dams and water diversions) on fish movement is estimated to be greater than that of large dams due to their high numbers (Januchowski-Hartley *et al*., 2013). Culverts allow for continued water connectivity below road-crossings but generally at greater velocities than the natural waterway because of a reduced cross-sectional area, creating hydraulic barriers (Ead *et al*., 2002; Norman *et al*., 2009). Culverts account for the majority of hydraulic barriers in developed waterways (Williams and Watford, 1997; Bouska and Paukert, 2010) and were originally designed to maximize water transport with little consideration of fish access (Warren and Pardew, 1998).

Design criteria of culverts have been revised in recent years to improve fish passage (Barnard *et al*., 2015; Van der Ree *et al*., 2015; Duguay and Lacey, 2016) but many existing structures require remediation (Andersen *et al*., 2012). While there are numerous remediation approaches, the effectiveness of culvert roughening (i.e. replacing smooth concrete substrates with rough, naturalistic substrates such as river stones and plants) has received little investigation (Newbold and Kemp, 2015; Goerig *et al*., 2016). Culvert roughening is predicted to improve fish passage by two mechanisms: via lowering the energetic cost of swimming and by increasing behavioural attraction to a more naturalistic structure. Roughened culverts have altered hydraulic properties so that reduced-velocity zones (RVZs) are created along the structure's base and walls (Powers *et al*., 1997; Richmond *et al*., 2007). Fish are hypothesized to utilize RVZs during passage, thereby lowering energetic costs (i.e. RVZ hypothesis; Powers *et al*. 1997; Richmond *et al*., 2007; Johnson *et al*. 2012). Support for the RVZ hypothesis has been documented in several species but these studies used corrugated metal substrates to increase roughness (Richmond *et al*., 2007; Johnson *et al*., 2012; Clark *et al*., 2014). Recent comparisons of pebbled and smooth substrates found no derived benefits to the swimming performance of juvenile shortnose sturgeon, *Acipenser brevirostrum* (Downie and Kieffer, 2017). This lack of energetic advantage was attributed to a small pebble size, relative to fish body size (Downie and Kieffer, 2017). The RVZ hypothesis remains untested for larger, naturalistic substrates, such as river stones. In addition to creating RVZs, roughening can also increase hydrodynamic heterogeneity (i.e. turbulence, Richmond *et al*., 2007). Turbulent flows are characterized by a mosaic of constantly fluctuating water speeds that fish may take advantage of, by timing swimming efforts with pockets of low-velocity or exploiting eddies to facilitate propulsion; a strategy termed kármán gaiting (Liao *et al*., 2003; Liao and Cotel, 2013).

Remediation approaches can be economically costly, with finite funds directed towards waterway restoration, deeming it imperative to ensure restoration efforts benefit target species. The aim of this study was twofold: (i) to determine if fish swimming performance is improved above rough compared to smooth substrates, and (ii) to model and evaluate the effectiveness of substrate roughening as a remediation strategy. Two small-bodied (<12 cm, total length, BL), freshwater species were used to address these aims: purple-spotted gudgeon, *Mogurnda adspersa* (Castelnau, 1878), and crimson-spotted rainbowfish, *Melanotaenia duboulayi* (Linnaeus, 1758). These species are sympatric and endemic to Australia, with populations spread along coastal catchments in south-east Queensland and northern New South Wales (Australian Conservation Agency, 1993). Both species are potamodromous, migrating within freshwater, and have experienced severe population declines in association with waterway development and fragmentation (Pusey *et al*., 1993; Boxall *et al*., 2002; Faulks *et al*., 2008; Carvalho *et al*., 2012). The purple-spotted gudgeon is listed as an endangered species under the ‘Fisheries Management Act, 1994’ in New South Wales, Australia, and is the focus of ongoing conservation initiatives. Small-bodied species were selected as this group is underrepresented in fish passage research, with the focus generally towards large, strong-swimming, iconic, recreational or commercial species (Pearson *et al*., 2006; Lacey *et al*., 2012). We predicted that: (H_1_) swimming performance would be markedly improved over rough compared to smooth substrates, and (H_2_) culvert remediation models would show roughening to be an effective approach, exemplified by higher maximum water velocities allowing successful upstream passage of fish.

## Materials and methods

### Fish maintenance

Crimson-spotted rainbowfish (*Melanotaenia duboulayi*) (*n* = 60; BL: mean ± s.d. 5.93 ± 0.9 cm; range 4.2–8.7 cm) and purple-spotted gudgeon (*Mogurnda adspersa*) (*n* = 60; BL: mean ± s.d. 9.99 ± 0.8 cm; range 7.71–11.58 cm) were obtained from a commercial hatchery (Australian Native Fish Enterprises, Burpengary, Queensland, Australia). Fish were housed in 45 L glass aquaria (*L *×* W *×* H*, 60 × 30 × 30 cm) at a stocking density of approximately 3 g (body mass) L^−1^ (*Mo. adspersa*) and 1 g L^−1^ (*Me. duboulayi*). Aquaria contained Brisbane city tap water conditioned with water primer (Prime^®^, Seachem, Georgia, USA), maintained at a constant temperature (25 ± 1°C). Water chemistry (pH, nitrogen and ammonia) was monitored weekly to ensure water quality. Fish were fed commercially supplied food pellets (Hikari^®^ Tropical Micro Wafers and TTanked Tropical+ food pellets) daily to satiation. The photoperiod was set to a 12-h light: 12-h dark cycle.

### Substrate design

**Figure 1:**
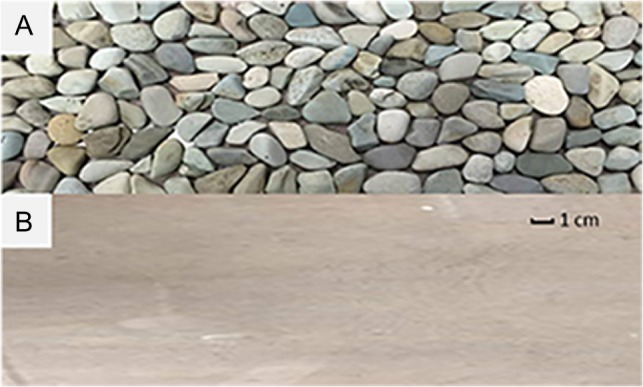
Substrates used in the swimming trials: (**A**) roughened substrate with fixed river stones and (**B**) smooth acrylic panel; River stones varied in shape and size but the majority were ~3 × 2 × 1 cm; *l* × *w *× *h*; River stone length equated to 0.3 BL and 0.5 BL for purple-spotted gudgeon (*Mo. adspersa*) and crimson-spotted rainbowfish (*Me. duboulayi*), respectively.

Swimming trials were conducted in a 185 L flow-controlled hydraulic flume (Loligo^®^, Tjele, Denmark; swim chamber dimensions: *L* ×*W* × *H*, 87 × 25 × 25 cm). Each swimming trial incorporated one substrate treatment, either a smooth acrylic panel or a custom-made, roughened substrate with fixed river stones (Fig. 1). River stones were glued to the acrylic panel in fixed positions to ensure each fish experienced the same conditions. The shape and size of river stones varied, but average stone diameter equated to 0.25 body lengths (BLs) and 0.40 BL for purple-spotted gudgeon (*Mo. adspersa*) and crimson-spotted rainbowfish (*Me. duboulayi*), respectively. The top surface area (SA) of each stone was measured using the particle analysis function in ImageJ (Schneider *et al*., 2012; median SA (SA_50_) = 4.99 cm^2^; median diameter (D_50_) = 2.52 cm) (see Supplementary Figs S1–S2). The substrates lined the bottom of the swim chamber (87 × 25 × 1.5 cm; *L *×* W *×* H*) and the swim chamber walls were made of smooth acrylic in both treatments. The substrates were detachable so treatment order could be randomized.

### Swim chamber calibration

**Figure 2:**
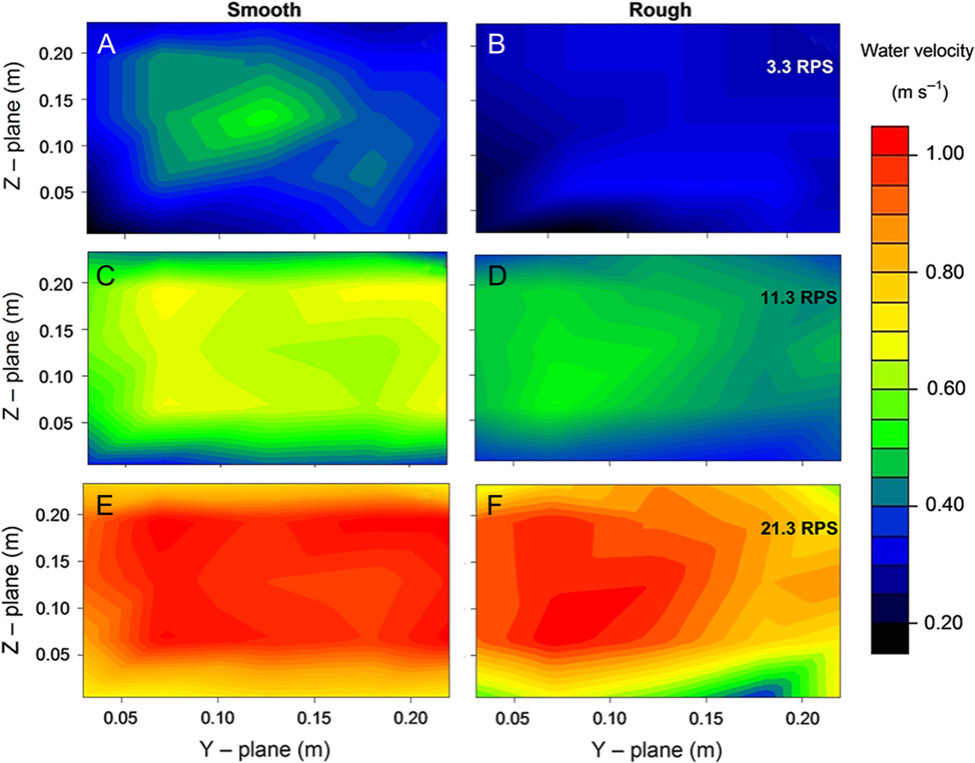
Water velocity (m s^−1^; represented by colour) heat maps of the swim chamber with smooth (left hand panel) and rough (right hand panel) substrates at three separate propeller speeds: 3.3 (**A**, **B**), 11.3 (**C**, **D**) and 21.3 (**E**, **F**) RPS (revolutions s^−1^); A 5 × 5 cross-section in the centre of the swim chamber was calibrated along the *Y*- and *Z*-planes, using a Pitot tube and custom-built air-water manometer. Time-averaged water velocities above the rough substrate were consistently lower than the smooth substrate at the same propeller speed.

**Figure 3:**
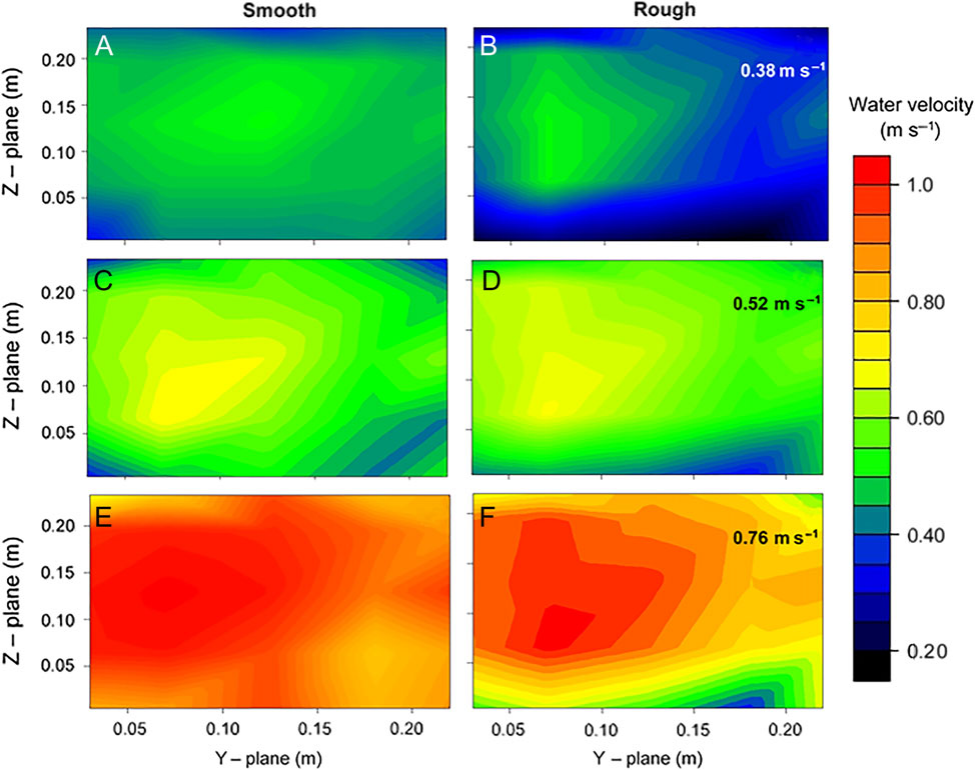
Water velocity (m s^−1^, represented by colour) heat maps of the swim chamber with smooth (left hand panel) and rough (right hand panel) substrates at a range time-averaged velocities (i.e. 0.38 m s^−1^**A**–**B**, 0.52 m s^−1^**C**–**D**, 0.76 m s^−1^**E**–**F**); A 5 × 5 cross-section in the centre of the swim chamber was calibrated along the *Y*- and *Z*-planes, using a Pitot tube and custom-built, air-water manometer; Hydrodynamic heterogeneity was increased and a greater number of RVZs were visualized along the base of the swim chamber in the rough compared to the smooth treatment.

The swim chamber was calibrated using a Pitot tube (Dwyer series 166, diameter = 3.18 mm, Dwyer Instruments^®^, Unanderra, AUS) and custom-built air-water manometer set to a 30° angled incline. A 5 × 5 cross-section in the centre of the swim chamber was measured for each water velocity increment (revolutions s^−1^) on the smooth and roughened substrates. A time-averaged water velocity (m s^−1^) calibration curve was determined for each substrate. The calibration curve for the smooth and roughened substrates were described by the following equations:

(1) $$V_{\mathrm{smooth}}=0.0382\mathrm{RPS}+0.0578;r^{2}=0.99,$$

(2) $$V_{\mathrm{rough}}=0.0392\mathrm{RPS}+0.0096;r^{2}=0.99,$$

where $V_{\mathrm{smooth}}$ and $V_{\mathrm{rough}}$ represent the time-averaged water velocity (m s^−1^) for the swim chamber containing smooth and rough substrates, respectively, and RPS represents the swimming flume's propeller speed (revolutions s^−1^). The rough substrate consistently derived lower water velocities for a given propeller speed compared to the smooth substrate between 6–18 revolutions s^−1^, and water velocity converged for both substrates at 21 revolutions s^−1^ (Fig. 2). Heat maps displaying the distribution of water velocity for both substrates at identical time-averaged velocities show increased hydrodynamic heterogeneity and a greater number of RVZs along the base of the swim chamber in the rough compared to the smooth treatment (Fig. 3).

### Swimming performance

Fish were tested individually in a post-absorptive state (fasted for 24 h). Substrate treatment was randomly assigned to fish using a random number generator (random.org; even number = smooth, odd number = rough), and fish were tested once to avoid training effects (Davison, 1997) (*n* = 30 per treatment, per species). Body sizes of fish were similar between rough and smooth substrate treatment groups for both species (*Mo. adspersa* BL mean ± s.d.; smooth 10.0 ± 0.8 cm, rough 9.9 ± 0.9 cm; *Me. duboulayi* smooth 6.1 ± 1.0 cm, rough 5.8 ± 0.8). Fish were allowed 30 min to adjust to conditions in the swimming flume with water velocity switched off (i.e. 0.00 m s^−1^), after which water velocity was increased every 5 min at increments of 0.05 m s^−1^, starting at 0.05 m s^−1^, until the fish fatigued. Fatigue was defined as the fish resting on the back wall of the swim chamber for ≥ 3 s. Total swimming time until fatigue and water velocity at fatigue were recorded to calculate critical swimming speed (*U*_crit_), using Brett's (1964) equation, as follows:

(3) $$U_{\mathrm{crit}}=U_{\begin{array}{c} f \end{array}}+\left[U_{i}\left(T_{f}/T_{i}\right)\right],$$

where $U_{\begin{array}{c} f \end{array}}$ is the highest velocity sustained for an entire 5 min interval (m s^−1^), $U_{i}$ is the water velocity increment (0.05 m s^−1^), *T*_*f*_ is the time (s) swum in the final increment and $T_{i}$ is the time interval (300 s). *U*_crit_ tests provided a measurement of the maximum velocity at which a fish can sustainably swim without fatiguing (Hammer, 1995; Peake, 2004) and were used to inform traversable water models. The 5 min increments in water velocity were of suitable duration, as weak swimming fishes can, in theory, travel 90 m upstream in 5 min (i.e. *U*_crit_ = 0.3 m s^−1^ × 300 s = 90 m)—a distance far exceeding the length of most culverts (Williams and Watford, 1997). Critical swimming speed measurements were standardized for fish body size, in terms of total body length per second (BL s^−1^), and both absolute (i.e. m s^−1^) and relative critical swimming speeds (BL s^−1^) are reported. The swim chamber was constantly aerated and water temperature was maintained at 25 ± 0.5°C using a submersible heater (300W Aqua One^®^, Ingleburn, Australia). Swimming gait was observed and classified as either direct, body-caudal fin (BCF) (Webb, 1998) or station-holding (Webb, 1989) where pectoral fins were used to grasp/hold position on the substrate. Measures of critical swimming speed continued when fish exhibited station-holding behaviour (Deslauriers and Kieffer, 2012; Kieffe *et al*., 2009). Total BL measurements were included in all analyses as a covariate. Following the completion of swimming trials, fish were lightly anaesthetized using AQUI-S (20 mg mL^−1^), blot-dried and photographed. ImageJ (National Institutes of Health, Maryland, USA) was used to measure BL for each fish. Cross-sectional body area of all fish was less than 10% of the cross-sectional area of the swimming flume chamber; therefore, corrections for the solid-blocking effect (Bell and Terhune, 1970) were not necessary.

### Culvert remediation models

To evaluate the effectiveness of culvert roughening, changes in swimming performance observed in the laboratory were assumed to translate to the field. Maximum traversable water velocities were modelled for both species swimming upstream through a range of culvert sizes (2–60 m in length), with either smooth or roughened substrates, using Peake *et al*.’s (1997) equation:

(4) $$Vf=Vs–\left(d\times {E_{\mathrm{Vs}}}^{-1}\right),$$

where Vf is the traversable water velocity (m s^−1^) within a culvert, Vs is the average critical swimming speed (*U*_crit_), *d* is the length of the culvert (m) and $E_{\mathrm{Vs}}$ is the endurance of the fish swimming at *V*_*s*_ (s). The endurance was 5 min, as that was the period of time in which the fish swam for before the velocity was increased in the swimming performance trials. Equation (4) acknowledges fish must swim faster than oncoming water velocity to achieve positive ground.

### Statistical analyses

Data analyses were performed using R Studio (version 3.1.3; R Core Team, 2012) using the MASS package (Venables and Ripley, 2002). The effect of substrate (two-level factor; rough/smooth) on swimming performance (*U*_crit_, m s^−1^) was determined using a one-way analysis of covariance (ANCOVA), with body size (BL) and holding tank number included as a covariates. Minimal adequate models were determined using stepwise simplification, and separate models were run for each species. *P*-values < 0.05 were considered statistically significant and all data are presented as mean ± s.e.

## Results

### Effect of substrate on swimming performance

**Figure 4:**
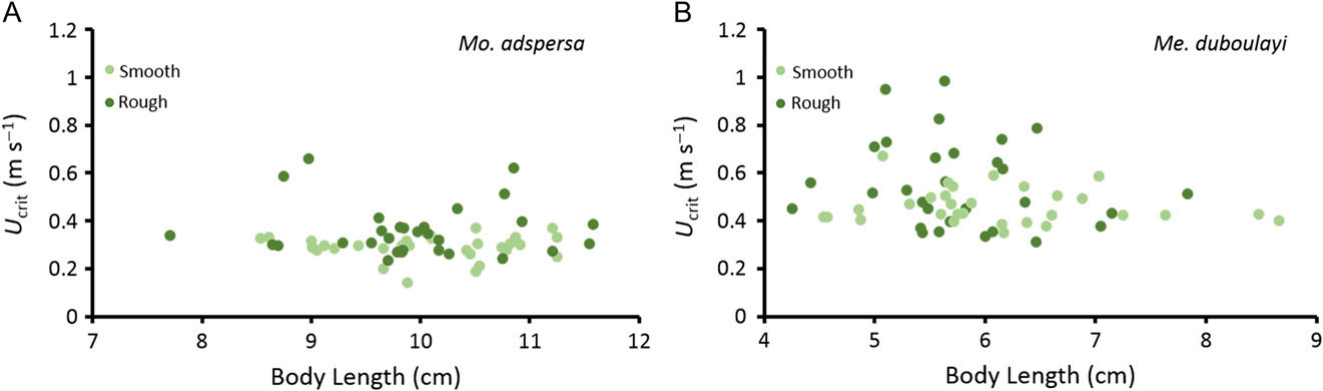
Effect of substrate type (i.e. smooth-light green circles, rough-dark green circles) on swimming performance (critical swimming speed, *U*_crit_, m s^−1^) of (**A**) purple-spotted gudgeon (*Mo. adspersa*) and (**B**) crimson-spotted rainbowfish (*Me. duboulayi*); Swimming performance was improved on rough compared to smooth substrates in both species (*P* < 0.05; ANCOVA; *n* = 30 treatment^−1^), and independent of BL (*P* ≥ 0.29; ANCOVA; *n* = 30 treatment^−1^); Values are shown as individual data points.

Substrate treatment had a significant effect on critical swimming speeds (*U*_crit_) of both *Me. duboulayi* (ANCOVA, *U*_crit_ = F_2, 57_ = 3.72, *P* < 0.05) and *Mo. adspersa* (ANCOVA, *U*_crit_ = F_2, 57_ = 5.21, *P* < 0.01) (Fig. 4). Swimming performance was markedly improved in the presence of the rough substrate, with *U*_crit_ increasing by 26.1% and 26.5% in *Me. duboulayi* (*U*_crit-smooth_ = 0.46 ± 0.01 m s^−1^, 7.79 ± 0.33 BL s^−1^ mean ± s.e.; *U*_crit-rough_ = 0.55 ± 0.03 m s^−1^, 9.83 ± 0.67 BL s^−1^ mean ± s.e.) and *Mo. adspersa* (*U*_crit-smooth_ = 0.28 ± 0.0 m s^−1^, 2.89 ± 0.1 BL s^−1^, mean ± s.e.; *U*_crit-rough_ = 0.36 ± 0.02 m s^−1^, 3.66 ± 0.22 BL s^−1^, mean ± s.e), respectively (Fig. 4). Critical swimming speed was independent of BL and holding tank number in both species (BL *P* ≥ 0.29; holding tank number *P* ≥ 0.17) and tank numbers were excluded from minimal adequate models. *Mo. adspersa* employed a combination of both station-holding and direct, BCF gaits in trials. *Me. duboulayi* employed direct, BCF gait in all trials but did not station-hold.

### Culvert remediation models

**Figure 5:**
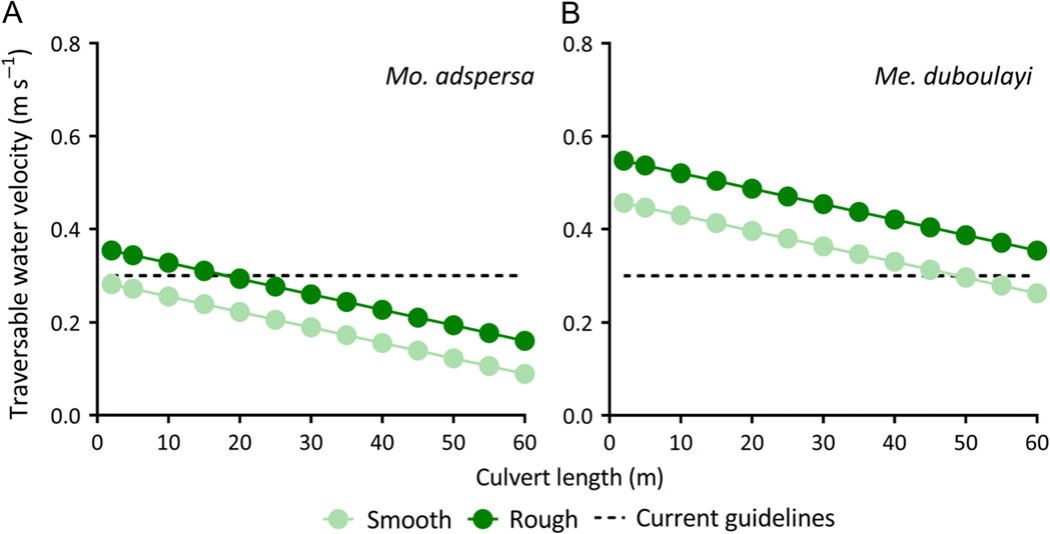
Modelled traversable water velocities (m s^−1^) allowing upstream passage of (**A**) purple-spotted gudgeon (*Mo. adspersa*) and (**B**) crimson-spotted rainbowfish (*Me. duboulayi*), through culverts (2–60 m in length) with rough and smooth substrates; Traversable water velocities are estimated to be higher for culverts with rough compared to smooth substrates; Horizontal dotted lines marks recommended water velocity limits (0.3 m s^−1^) in Australia (New South Wales).

Culvert remediation models predicted maximum water speeds allowing successful upstream passage of both species to decrease with increasing culvert length, across the range of 2–60 m (Fig. 5). Maximum water velocities enabling upstream movements varied depending on substrate type, with allowable velocities markedly lower in culverts with smooth compared to rough substrates (Fig. 5). To enable upstream movements of *Me. duboulayi* through ‘small’ (2 m), ‘medium’ (<20 m) and ‘large’ (20 ≤ 60 m) culverts with a smooth substrate, water velocities would need to be ≤0.46, 0.40 and 0.26 m s^−1^, respectively (Fig. 5B). However, these water velocities were predicted to increase to ≤ 0.55, 0.49 and 0.35 m s^−1^ for the same sized culverts (i.e. small, medium and large) if culvert interiors were roughened (Fig. 5B). Similarly, to enable upstream movement of *Mo. adspersa* through ‘small’, ‘medium’ and ‘large’ smooth culverts, water velocities would need to be as low as ≤0.28, 0.22 and 0.09 m s^−1^, respectively; whereas these velocities increase to ≤ 0.35, 0.29 and 0.16 m s^−1^ in roughened culverts (Fig. 5A).

## Discussion

Roughened culverts are often assumed to improve fish swimming performance and upstream passage (Barnard *et al*., 2015), but empirical assessments are lacking. Introducing fixed river stones into the swim chamber concurrently reduced water velocity and created RVZs along the substratum. Substrate roughening improved relative swimming performance of *Me. duboulayi* amd *Mo. adspersa* by ~26%, supporting our original hypothesis (H_1_). This heightened performance translated into the traversable water velocity models, with maximal allowable water speeds being higher in roughened compared to smooth culverts, suggesting roughening may be an effective remediation approach to improve fish passage.

### Improved swimming performance: hydraulic factors

Swimming performance in the roughened treatment was likely improved due to altered hydraulics in the swim chamber. Roughening substrates can increase both the intensity of turbulence (i.e. turbulent kinetic energy; TKE) and the size and number of eddies generated (Papanicolaou and Talebbeydokhti, 2002; Nikora *et al*., 2003). Mosaics of fluctuating water velocities can be both detrimental and beneficial to fish swimming performance. High intensities of TKE can increase the energetic cost of swimming (Enders *et al*., 2005) and disorientate/unbalance fish (Tritico and Cotel, 2010). For example, a velocity-dependent cost was identified over pebbled substrates, whereby endurance was reduced and bottom-swimming behaviours were down-regulated at high speeds, suggesting stability was reduced compared to smooth substrates (May and Kieffer, 2017). Alternatively, low intensities of TKE can improve swimming performance if fish exploit low-velocity zones (Powers *et al*., 1997; Johnson *et al*., 2012). Juvenile Coho Salmon (*Oncorhynchus kisutch*), for instance, have been observed to actively exploit reduced-velocity pathways during upstream movement through a culvert test bed (Johnson *et al*., 2012). Improved swimming performance in the rough treatment here suggests the river stones generated a beneficial level of turbulence which may have lowered the energetic cost of swimming and extended endurance, but further experimentation is required for confirmation. The river stones may have further altered hydraulic conditions by generating a greater number of vortices/eddies (Papanicolaou and Talebbeydokhti, 2002; Nikora *et al*., 2003). Fish can harness energy from vortices to facilitate forward propulsion and reduce energy expenditure (i.e. kármán gaiting, Liao *et al*., 2003; Liao and Cotel, 2013). The next progression would be to measure the metabolic cost of transport of fish swimming above roughened and smooth substrates.

### Swimming gaits

*Mo. adspersa* and *Me. duboulayi* were similarly affected by the rough substrate, with both species experiencing a ~26% increase in swimming performance, despite different gaits employed during swim trials. *Mo. adspersa* employed station-holding behaviour in all trials, whereas *Me. duboulayi* did not station-hold and instead, employed a BCF swimming mode. Species utilizing bottom-swimming behaviours (e.g. station-holding and substratum-skimming) are expected to derive a greater net benefit from substrate roughening than fishes reliant on BCF modes, as these energy-saving behaviours are largely ineffective on smooth surfaces (Kieffer *et al*., 2009). Bottom-swimming behaviours are increased at intermediate velocities over rough compared to smooth substrates in a number of species (Adams *et al*., 2003; May and Kieffer, 2017). The behaviours underpinning improved performance here remain unidentified and fine-scale behavioural trials are required to determine if station-holding or kármán gaiting are altered by varied combinations of substrate treatments and water velocities. It is likely that *Mo. adspersa* and *Me. Duboulayi* benefited from the roughened substrate in different ways but it is clear that roughening can improve swimming performance and energetics of species with disparate morphologies and behaviours.

### Implications for culvert remediation and design

Successful passage through culverts is critically important as population declines of both *Me. duboulayi* and *Mo. adspersa* have been linked to movement barriers (Boxall *et al*., 2002; Hattori and Warburton, 2003; Faulks *et al*., 2008; Carvalho *et al*., 2012; NSW DPI, 2013). In agreement with H_2_, culvert remediation models predict substrate roughening to improve passage of both species, with a roughened substrate allowing water velocities to be substantially higher than required for culverts with smooth substrates. Design recommendations in Australia (New South Wales) limit water velocities through culverts to a maximum of 0.3 m s^−1^. At this velocity maximum culvert transit is likely to be unrestricted for *Me. duboulayi* for culverts up to 50 m in length, but compromised for *Mo. adspersa* in culverts with smooth substrates. Passage is predicted to be restricted for *Mo. adspersa* in culverts 2–15 m in length with a smooth substrate (i.e. maximum allowable velocities 0.23–0.28 m s^−1^), but roughening remediation increases allowable water velocities to levels exceeding current guidelines (i.e. 0.31–0.35 m s^−1^). Transit through very long culverts (>20 m) is likely to be restricted for *Mo. adspersa* even with roughening remediation, and these structures may require additional restoration efforts, such as the installation of rest areas (Feurich *et al*., 2012). Implementing rough substrates in culverts could be a cost-effective and straight-forward approach to improving fish passage prospects, and far less difficult than engineering culverts that only allow for very low water velocities (e.g. <0.3 m s^−1^).

Outputs from our traversable water velocity models were similar to other small-bodied species (e.g. Mitchell, 1989 [flathead mullet, *Mugil cephalus*]; Doehring *et al*., 2011 [juvenile inanga, *Galaxias maculatus*]; Rodgers *et al*., 2014 [empire gudgeon, *Hypseleotris compressa*]). Similar to *Mo. adspersa*, many small-bodied species have been identified as weak-swimmers, requiring very low water velocities for upstream movements (ranging 0.05–0.20 m s^−1^). Reducing water velocity to this extent can be challenging, but culvert roughening may be a solution that allows hydraulic efficiency goals to be met without compromising fish access. Examining the effect of substrate roughening on the swimming performance of a greater number of species, with variations in morphology and swimming gaits, will allow us to gauge the potential benefit and wider application of roughening fish passes. In contrast to our findings, previous research has found roughening to provide no benefit to fish swimming performance (Newbold and Kemp, 2015). Newbold and Kemp (2015) found corrugated roughening of swim chamber walls to have no effect on the swimming performance of juvenile cyprinids (*Cyprinus carpio*); but this study differed to ours with respect to the position (i.e. walls compared to bottom of swim chamber) and type (i.e. corrugated inserts compared to river stones) of substrate. Roughening the walls of culverts/experimental swim chambers, compared to the bottom, likely differentially affects hydraulic conditions (e.g. level of TKE). Wall roughening has been suggested to generate detrimental levels of turbulence, where the energetic expense of swimming is increased and fish become disorientated/unbalanced (Newbold and Kemp, 2015). Wall roughening may be less effective at facilitating station-holding behaviour compared to substrate roughening, and may not benefit fishes reliant on this behaviour for upstream passage. Further studies examining how swimming performance is altered in response to variation in substrate size (e.g. rock diameter relative to fish size), substrate type (e.g. corrugate surfaces, river stones, concrete with a rough finish) and roughening position (e.g. walls, bottom or entire culvert interior) is warranted.

Although the culvert remediation model presented here can be a powerful tool for decision making, the limitations of this model need to be considered. Estimates of swimming performance derived from non-volitional, laboratory studies can underestimate true ability, as fish often attain greater swimming speeds in open-channel, volitional trials (Hinch and Bratty, 2000; Peake, 2004). The swimming performance data provided here are likely conservative estimates of true swimming ability and in situ validation of these findings is necessary. Nonetheless, our findings provide a baseline assessment of the effectiveness of culvert roughening, and strongly suggest that roughened substrates can improve fish swimming performance and potentially passage prospects. Remediation of existing culverts may have far-reaching benefits by reconnecting the aquatic environment.

## Supplementary Material

Supplementary Data

Supplementary Data

Supplementary Data
