# Supplementary material for: Substrate roughening improves swimming performance in two small-bodied riverine fishes: implications for culvert remediation and design
Source: Conserv Physiol. 2017 May 26;5(1):cox034. doi: 10.1093/conphys/cox034 (PMC5445438; doi:10.1093/conphys/cox034)
Supplement: Supplementary Data [file FigureS1.pdf]

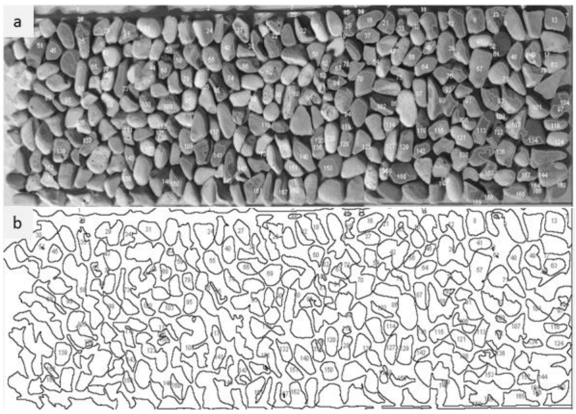

Figure S1: Roughened substrate profiling. Particle analysis was used to identify and measure individual stones on the roughened substrate (a), and imageJ generated a particle map (b) to calculate average stone surface area.
