# Supplementary material for: Substrate roughening improves swimming performance in two small-bodied riverine fishes: implications for culvert remediation and design
Source: Conserv Physiol. 2017 May 26;5(1):cox034. doi: 10.1093/conphys/cox034 (PMC5445438; doi:10.1093/conphys/cox034)
Supplement: Supplementary Data [file FigureS2.pdf]

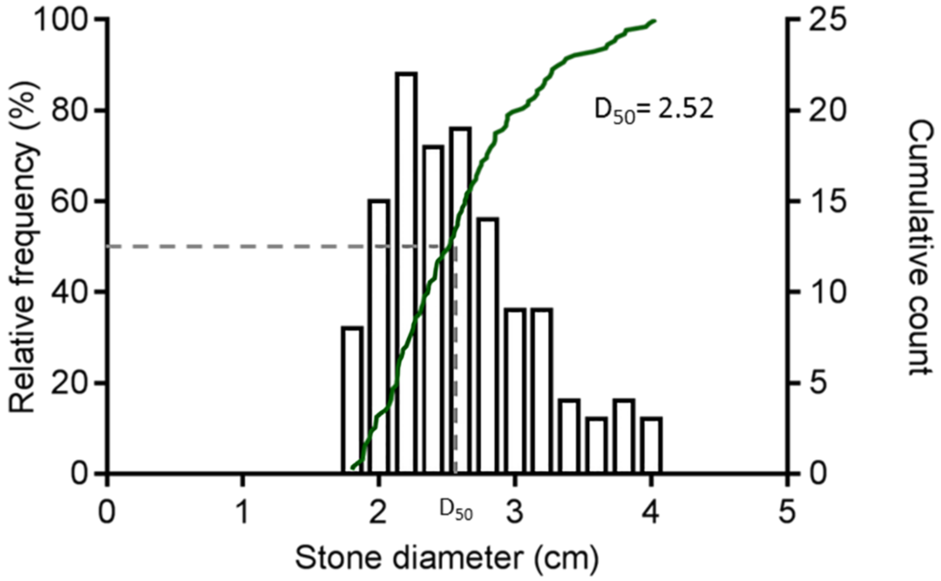

Figure S2: Histogram of relative frequency (%; left y-axis) and cumulative count (green line; right y-axis) of stone diameter. The grey dotted lines mark the calculation of the D_{50} (the rock size [diameter] of which 50 % of the rocks are smaller). The D_{50} for the experimental roughened surface was 2.52 cm, assuming circular rock shape.
