# Supplementary material for: Substrate roughening improves swimming performance in two small-bodied riverine fishes: implications for culvert remediation and design
Source: Conserv Physiol. 2017 May 26;5(1):cox034. doi: 10.1093/conphys/cox034 (PMC5445438; doi:10.1093/conphys/cox034)
Supplement: Supplementary Data [file Supplementarymaterial.docx]

**Supplementary Material**

*Roughened substrate profiling*

The substrate roughened with river stones was photographed (aerial view) in an evenly illuminated room. The application ImageJ (Schneider *et al.,* 2012) was used to analyse stone size on a fine scale. A bandpass filter was applied to the image and the threshold level was adjusted to separate individual stones. Particle analysis function was used to measure the top surface area (SA) of each stone. A total of 128 stones were outlined and measured (Fig. S1) and the median stone SA was 4.99 cm^2^ and median stone diameter (D_50_) was 2.52 cm assuming circular shape (Fig. S2).
